# Comparative proteomics analysis of *Schistosoma japonicum* developed in different *Oncomelania* snails as intermediate hosts

**DOI:** 10.3389/fcimb.2022.959766

**Published:** 2022-11-01

**Authors:** Gongzhen Liu, Feng Miao, Yongbin Wang, Jingxuan Kou, Kun Yang, Wei Li, Chunrong Xiong, Fengjian Zhang, Xinyao Wang, Haoyun Yan, Changyin Wei, Changlei Zhao, Ge Yan

**Affiliations:** ^1^ College of Agriculture and Forestry, Linyi University, Linyi, Shandong Province, China; ^2^ Shandong Institute of Parasitic Diseases, Shandong First Medical University & Shandong Academy of Medical Sciences, Jining, Shandong Province, China; ^3^ Jiangsu Institutes of Parasitic Diseases, Wuxi, Jiangsu Province, China; ^4^ Fourth Hospital of Weishan, Jining, Shandong Province, China; ^5^ Shandong Weishan Center for Disease Prevention and Control, Jining, Shandong Province, China

**Keywords:** *Schistosoma japonicum* (*S. japonicum*), schistosomiasis, isobaric tags for relative and absolute quantification (iTRAQ), differentially expressed proteins (DEPs), *Oncomelania hupensis* (*O.hupensis*), *Oncomelania weishan* (*O.weishan*)

## Abstract

Schistosomiasis is a tropical parasitic disease that seriously endangers humans and animals. In this study, two *Oncomelania* snails, *Oncomelania hupensis* (*O. hupensis*) and *Oncomelania weishan* (*O. weishan*), were infected with *Schistosoma japonicum* (*S. japonicum*) cercariae during the early period, and ICR mice were subsequently infected with two kinds of miracidia that developed in male and female adult worms. In this study, isobaric tags for relative and absolute quantification (iTRAQ) were used to identify four channels: 113, 115, 117, and 119. A total of 2364 adult schistosome proteins were identified, and 1901 proteins were quantitative. Our results revealed 68 differentially expressed proteins (DEPs) in female adult worms, including 24 upregulated proteins and 44 downregulated proteins, and 55 DEPs in male adult worms, including 25 upregulated proteins and 30 downregulated proteins. LC-MS/MS and bioinformatics analysis indicated that these DEPs are mainly concentrated in cellular composition, molecular function, biological function and catabolism pathways. In summary, this proteomics analysis of adult schistosomes that hatched in two intermediate hosts helps to improve our understanding of the growth and developmental mechanisms of *S. japonicum*.

## Introduction

Schistosomiasis is an important zoonotic parasitic disease that is widely prevalent in tropical and subtropical countries and regions, seriously endangering human health and hindering social and economic development. World Health Organization data showed that 260 million people are infected with *S. japonicum*, and more than 700 million people are threatened worldwide (GBD 2016 Causes of Death Collaborators, 2017). With the implementation of effective prevention and control measures, schistosomiasis has been reduced or blocked significantly in endemic countries (Li et al., 2016). However, most developing countries, especially in Sub-Saharan Africa, have experienced additional spreading of schistosomiasis because of extreme poverty, inadequate medical conditions and insufficient knowledge (Li et al., 2016). *Schistosoma mansoni* (*S. mansoni*) is prevalent in Sub-Saharan Africa, Central and South America, while *Schistosoma haematobium* (*S. haematobium*) is prevalent in Africa and the Arabian Peninsula. *S. japonicum* is prevalent in China, the Philippines and Indonesia, while *Schistosoma mekongi* (*S. mekongi*) and *Schistosoma intercalatum* (*S. intercalatum*) are endemic in local areas (Vos et al., 2012).

Schistosomiasis has been endemic in China for more than 2170 years. The discovery of *S. japonicum* eggs in a female corpse from the Hunan Changsha Mawangdui site and a male corpse from Hubei Jiangling confirmed that schistosomiasis emerged as early as the West Han Dynasty of China (Song et al., 2016). Schistosomiasis is seriously epidemic in the Yangtze River Basin and the southern provinces and is a major parasitic disease that seriously endangers people’s health and affects economic development (Song et al., 2016). The transmission and epidemiology of schistosomiasis are affected by natural, social and biological factors. The prevention and control strategy for schistosomiasis is a comprehensive control strategy based on the control of infectious sources, including the elimination of infection sources, the control of the intermediate host, *Oncomelania* snails, improved management of human and livestock manure, the provision of safe water and health education. As the main host and infection source, cattle play an important role in the transmission of schistosomiasis.

Parasite invasion affects host cell expression at the transcript and protein levels. The host cell also exerts immune pressure on the parasite and affects parasite gene expression. Several reports have shown that the transcriptome and proteome of *S. japonicum* differ significantly in many hosts (Gobert et al., 2009; Hong et al., 2011; Han et al., 2012; Zhai et al., 2018). Differentially expressed genes from the different developmental stages of *S. japonicum* revealed a wide range of functions and processes, including host immune response, energy production, calcium signaling, sphingolipid metabolism and egg production (Gobert et al., 2009). A comparative characterization of miRNAs from *S. japonicum* grown in Wistar rats and BALB/c mice revealed four differentially expressed miRNAs, which were analyzed and involved in sexual maturation, embryo development in the schistosome and the pathogenesis of schistosomiasis (Han et al., 2015). In addition, a total of 131 DEPs were identified between water buffalo and yellow cattle by iTRAQ-coupled LC-MS/MS (Hong et al., 2011). Comparative proteomics analyses demonstrated that 21-39 protein spots were significantly differentially expressed in mice, rats and reed voles (Zhai et al., 2018).

Proteomics research has been widely applied in the field of viruses, bacteria and parasites. Proteomics can not only provide evidence for cell biology but also elucidate the molecular mechanisms of diseases. Proteomics investigates the differentially expressed proteins (DEPs) induced by physiological and pathological mechanisms, metabolic regulation, and protein expression profiles, revealing the response of an organism to internal and external environmental changes. Proteomics is widely applied to study eukaryotic parasites, such as *S. japonicum*. Previous proteomics research on *S. japonicum* used two-dimensional (2D) gel electrophoresis. In ultraviolet-attenuated and normal *S. japonicum* cercariae, 20 DEPs were identified by mass spectrometry (Yang et al., 2009). Compared with two-dimensional (2D) gel electrophoresis, iTRAQ is more suitable and sensitive for the comparison and identification of DEPs (Wu et al., 2006). iTRAQ-based analysis of adult *S. japonicum* from water buffalo and yellow cattle showed that 131 DEPs were primarily involved in protein synthesis, transcriptional regulation, protein proteolysis, cytoskeletal structure and oxidative stress response processes (Zhai et al., 2018). The results showed that *S. japonicum* development in different hosts leads to several DEPs (Zhai et al., 2018).

**Figure 1:**
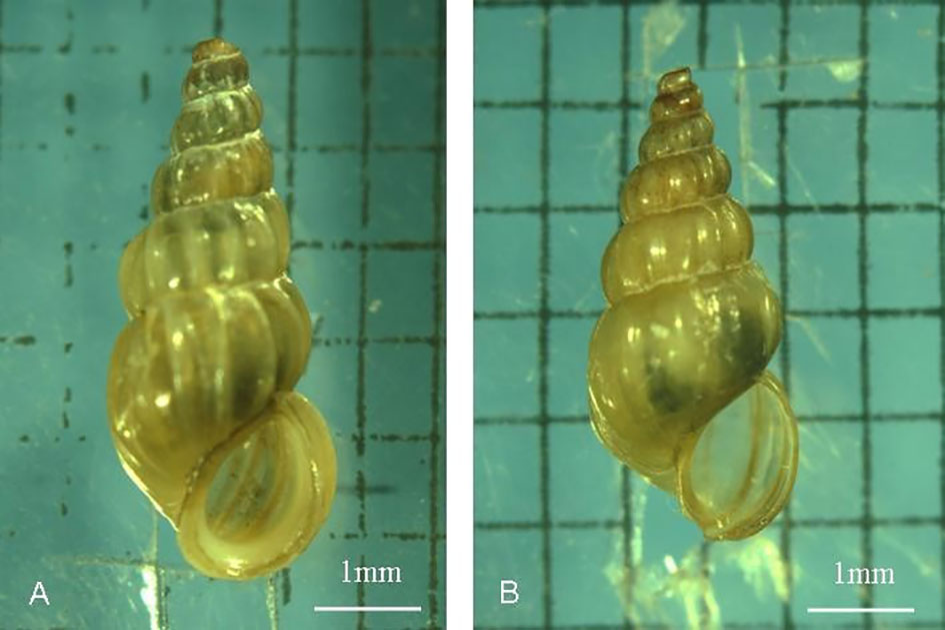
Comparison of shell shape changes after 12 generations of O. hupensis snails in the Weishan Lake region. Compared with the Jiangsu snails, the body size of the Shandong snails in the Weishan Lake region decreased, and their shells became thinner. **(A)** *(O. hupensis snails )*; **(B)** *(O. Weishan snails )*.

*O. hupensis* is the only intermediate host of *S. japonicum* (Zhou et al., 2007; Zhao et al., 2010). The northernmost distribution area of *O. hupensis* is located in Gaoyou County, Jiangsu Province (35.15 degrees north latitude) in China (Miao et al., 2014). In October 2004, adult *O. hupensis* specimens were collected from the Yangzhou section of the Changjiang River on the East Route of the South-North Water Diversion Project of China and kept in the southern foothills of Dushan Island in Weishan Lake, Shandong Province. After 13 years of breeding on Dushan Island and 12 generations of snails, the shell shape and population of the snails changed (Miao et al., 2014) (**Figure 1**). Therefore, we tentatively named the snails *Oncomelania weishan (O. weishan)* in this research. During more than ten years of evolution, the morphology of *O. weishan* evolved distinctly. If the miracidia complete their development through the natural environment process in the screw body, escape from the infected cercariae and invade mice, it will be of great significance to study the biology and life cycle of *S. japonicum* in *O. weishan*.

However, little research has been conducted on the proteome of adult *S. japonicum* specimens during development in two species of intermediate hosts, *O. hupensis* and *O. weishan*. 

In this study, we aimed to analyze the proteomic changes in female and male adult *S. japonicum* worms that developed in *O. hupensis* and *O. weishan* intermediate *Oncomelania* snails. The iTRAQ assay was used to analyze the differential proteomic profiles between female and male adult schistosomes. The total results showed that the regulation of DEPs will provide invaluable information about the metabolic pathways and development of *S. japonicum.*

## Results and discussion

**Figure 2:**
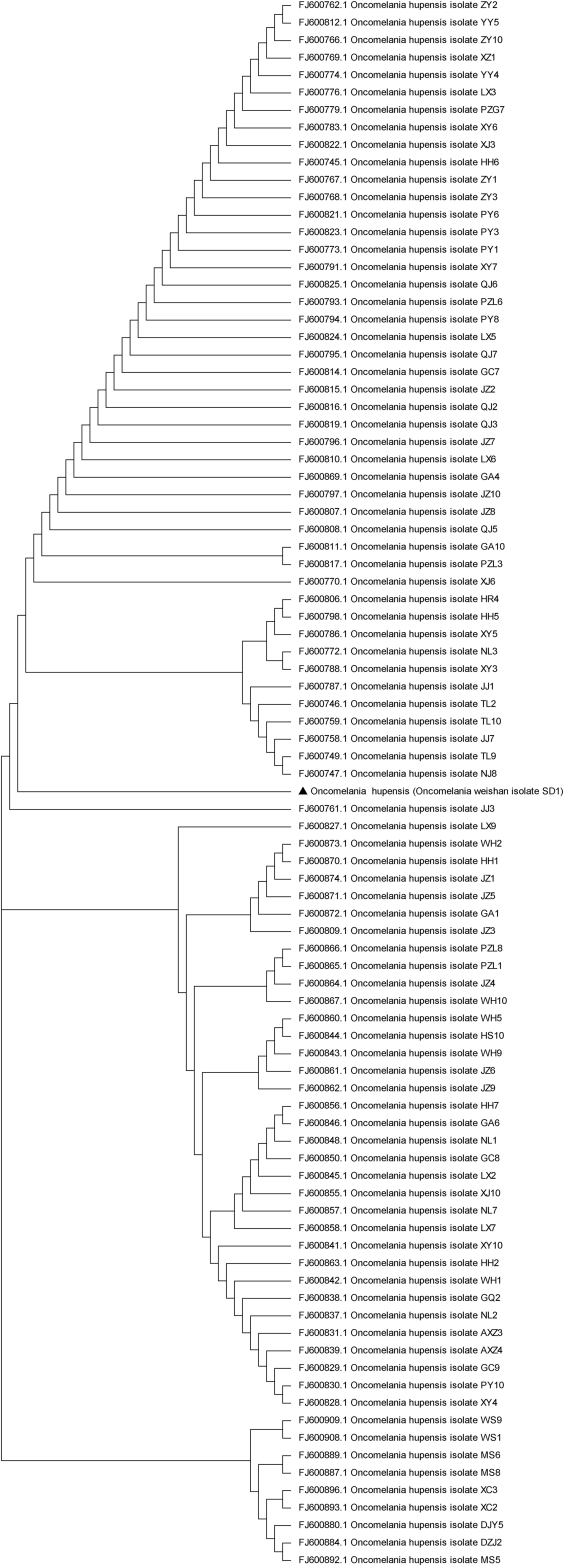
Genetic evolution analysis of *O.weishan* isolate SD1.

Schistosomiasis, which is caused by *S. japonicum*, *S. mansoni* and *S. egyptian*, is a zoonosis that seriously endangers human health and animal husbandry (Vos et al., 2012). *O.hupensis* is the only intermediate host of *S.japonicum* and plays a key role in the transmission of schistosomiasis. *O. hupensis* is mainly distributed in the Yangtze River Valley and the southern region of China. Previous reports have shown that the development of schistosomes differs significantly in different hosts (Yang et al., 2012). The biological characteristics between *O.hupensis* and *O.weishan* didn’t showed apparently variety. Compared with those of the *O. hupensis* snails, the most difference was the body size of the *O. weishan* snails in the Weishan Lake region decreased, and their shells became thinner (**Figure 1**); Meanwhile, *O. weishan* snails can adapt to the low temperature environment in the Shandong province, *O. hupensis* snails can not adapt in north. We cannot exclude the possibility that environmental factors affect snail breeding, which leads to a natural tendency toward decay over time. The amplified products PCR amplification of the conserved 5.8S gene sequence of *O.hupensis* and *O.Weishan*, the gene sequencing and evolutionary tree confirmed *O.Weishan* showed some difference with *O.hupensis* (**Figure 2**). Homology analysis of gene evolution tree results showed that *O.weishan* isolate SD1 near *O.hupensis* isolate NJ8 and *O.hupensis* isolate TL9 (Zhao et al., 2010).

**Figure 3:**
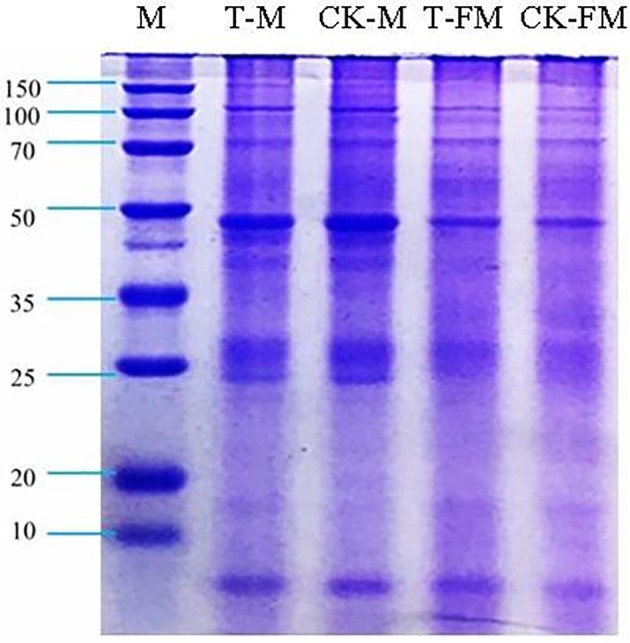
SDS-PAGE of adult schistosome proteins. T-M: Male adult schistosomes (*O. weishan*); CK-M: Male adult schistosomes (*O. hupensis*); T-FM: Female adult schistosomes (*O. weishan*); CK-FM: Female adult schistosomes (*O. hupensis*).

The iTRAQ technique in combination with LC-MS/MS showed that the protein expression profiles of adult worms also differed between yellow cattle and water buffalo (Zhai et al., 2018). We analyzed the four groups of adult schistosome proteins by SDS-PAGE. Each sample exhibited distinct band patterns and was subsequently prepared for LC-MS/MS analysis (**Figure 3**), however, we still found some tiny difference between T_FM/CK_FM group and T_M/CK_M group.

**Figure 4:**
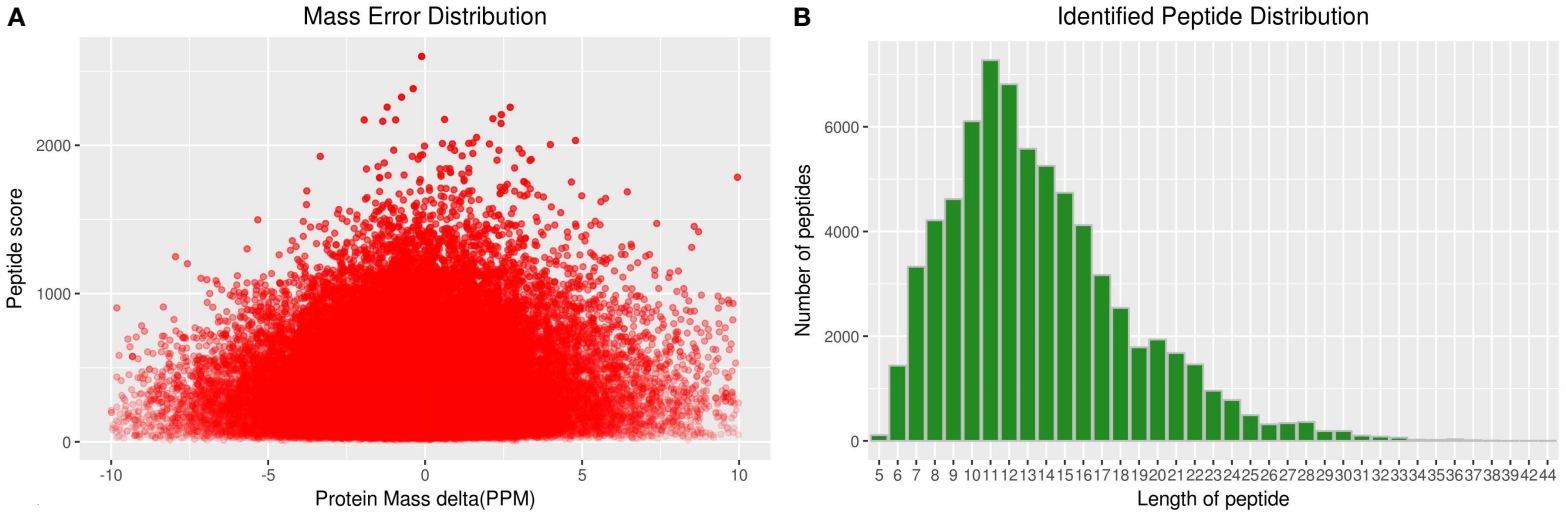
Total identified protein distribution of adult schistosomes.

**Figure 5:**
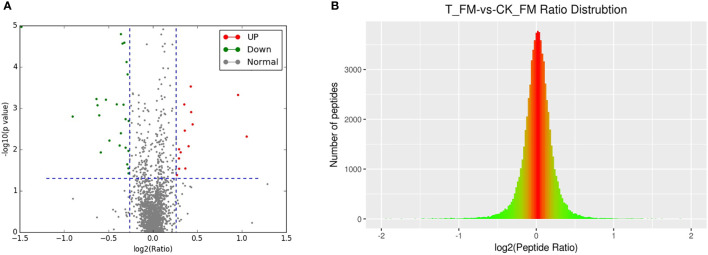
DEP distribution ratio of female adult schistosomes.

**Figure 6:**
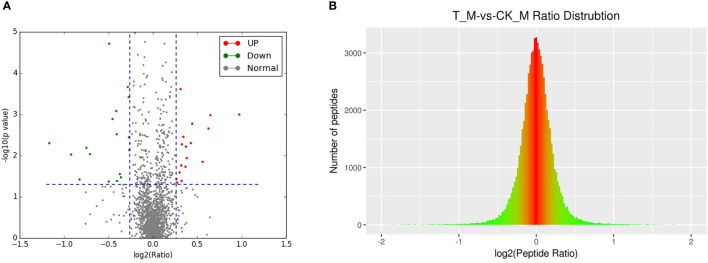
DEP distribution ratio of male adult schistosomes.

A total of 2364 adult schistosome proteins were identified, and 1901 proteins were quantitative (**Figure 4**). Our results showed 68 DEPs in female adult worms, including 24 upregulated proteins and 44 downregulated proteins in the T_FM/CK_FM group, and 55 DEPs in male adult worms, including 25 upregulated proteins and 30 downregulated proteins in the T_M/CK_M group. The DEP distribution ratios obtained from our analysis are displayed in **Figure 5** and **Figure 6**. DEPs were defined based on threshold protein quantification (1.2 & 0.05).

**Figure 7:**
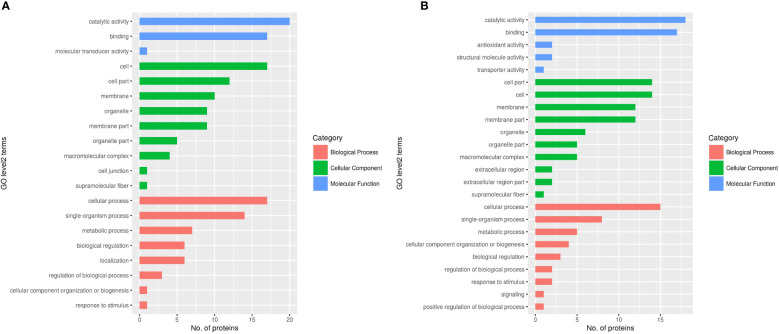
GO analysis of DEPs in female and male adult schistosomes. **(A)** Female; **(B)** Male.

After the analysis of DEPs, cellular component, molecular function, biological progress and subcellular location were used to classify the DEPs by GO analysis. Sixty-eight DEPs from female worms are involved in nine cellular components, including 32 upregulated proteins and 36 downregulated proteins (**Figure 7A**). In addition, 73 DEPs from male worms are involved in the composition of ten cellular components, including 25 upregulated proteins and 48 downregulated proteins (**Figure 7B**). According to the results, there are more downregulated proteins in male worms than female worms.

Thirty-eight DEPs from female worms are involved in three molecular functions (binding, molecular transducer activity and catalytic activity), including 19 upregulated proteins and 19 downregulated proteins (**Figure 7A**). Seventeen DEPs are involved in binding, 20 DEPs are involved in catalytic activity, and one protein is involved in molecular transducer activity. In addition, 40 DEPs from male worms are involved in five molecular functions (binding, structural molecule activity, transporter activity, antioxidant activity and catalytic activity), including 16 upregulated proteins and 14 downregulated proteins. Seventeen proteins are involved in binding, and 18 proteins are involved in catalytic activity. Two proteins are involved in structural molecule activity, two proteins are involved in antioxidant activity, and one protein is involved in transporter activity (**Figure 7B**). The data showed that there are obviously more kinds of molecular functional DEPs in male worms than in female worms. The common similarity of DEPs was the binding in molecular functions between male worms and female worms. Fifty-two DEPs from female worms are involved in eight biological processes (localization, biological regulation, metabolic process, response to stimulus, cellular component organization or biogenesis, cellular process, and single-organism process), including 30 upregulated proteins and 22 downregulated proteins (**Figure 7A**). Forty-one DEPs from male worms are involved in nine biological processes (biological regulation, metabolic process, response to stimulus, cellular component organization or biogenesis, positive regulation of biological process, cellular process, signaling and single-organism process), including 13 upregulated proteins and 28 downregulated proteins (**Figure 7B**). The number of biological processes proteins of female worms is obviously more than that of male worm strains.However, there are obviously more kinds of DEPs in male strains than in female strains including signaling and positive regulation of biological process.

**Figure 8:**
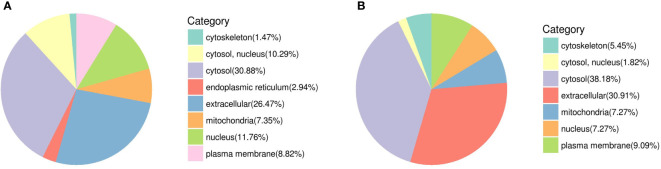
DEP category distribution for subcellular location. **(A)** Female; **(B)** Male.

Sixty-eight DEPs from female worms are involved in eight locations (**Figure 8A**) (cytosol and nucleus (10.29%), cytosol (30.88%), endoplasmic reticulum (2.94%), mitochondria (7.35%), nucleus (11.76%), cytoskeleton (1.47%), extracellular (26.47%), and plasma membrane (8.82%)), including 24 upregulated proteins and 44 downregulated proteins. Fifty-five DEPs from male worms are involved in seven locations (**Figure 8B**) (cytosol and nucleus (1.82%), cytosol (38.18%), mitochondria (7.27%), nucleus (7.27%), cytoskeleton (5.45%), extracellular (30.91%), plasma membrane (9.09%)), including 25 upregulated proteins and 30 downregulated proteins. The similarity of DEP category distribution between femal and male for subcellular location mainly concentrate in cytosol and extracellular (**Figures 8A, B**). Meanwhile, the obvious difference was the subcellular location of endoplasmic reticulum account for 2.94% in female worms.

**Figure 9:**
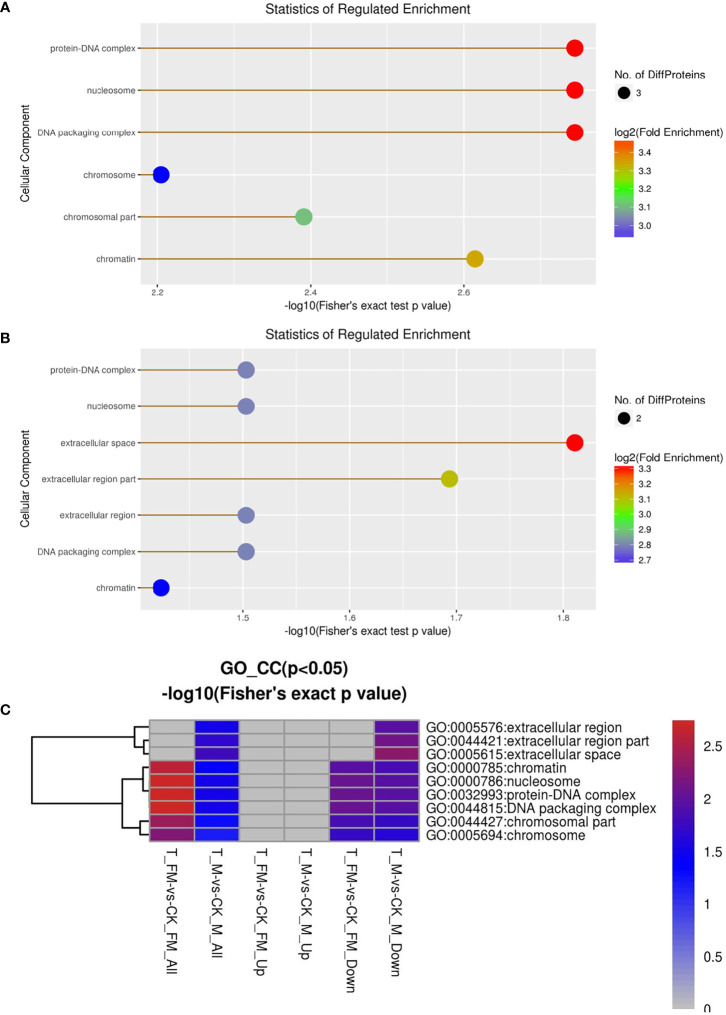
GO enrichment analysis for cellular component in female **(A)** and male **(B)** adult *S. japonicum* specimens.

Cellular component, molecular function, biological process and Kyoto Encyclopedia of Genes and Genomes (KEGG) were used to classify the DEPs by GO enrichment analysis. First, we calculated the GO enrichment of DEPs associated with cellular components, including protein-DNA complex, nucleosome, DNA packaging complex, chromosome, chromosomal part, chromatin, extracellular region and extracellular space (**Figures 9A, B**). The results revealed 25 DEPs in females and 27 DEPs in males among 934 proteins. Eleven DEPs each were upregulated in females and males, while 14 DEPs and 16 DEPs were downregulated in females and males, respectively (**Figure 9C**).

**Figure 10:**
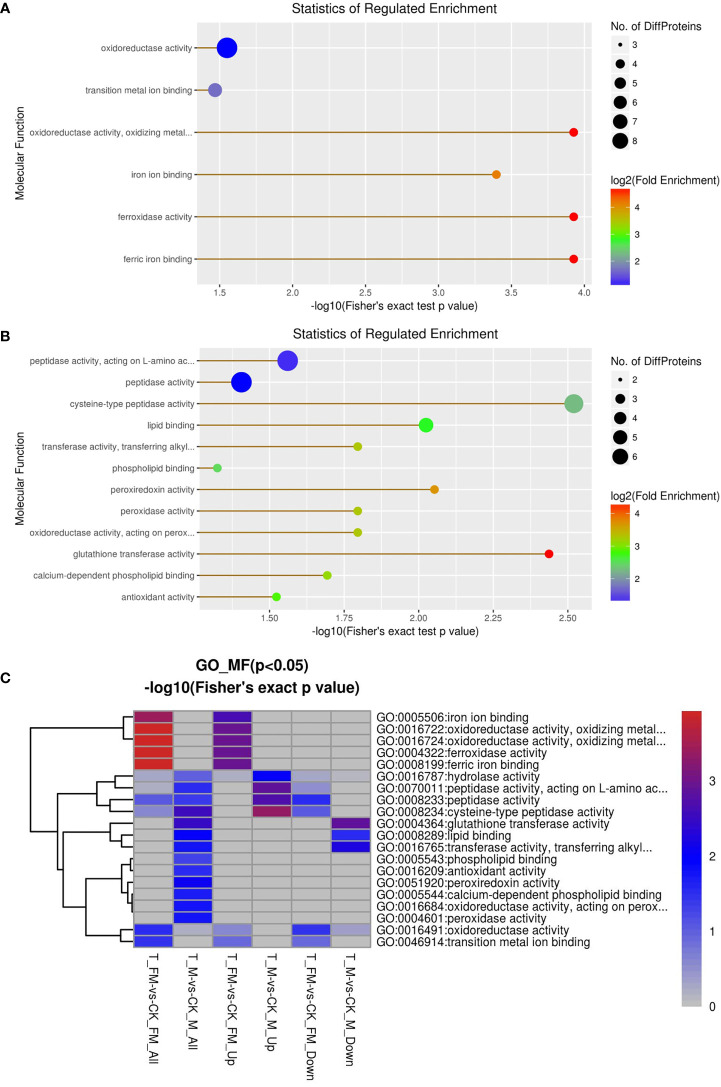
GO enrichment analysis for molecular function in female **(A)** and male **(B)** adult *S. japonicum* specimens.

**Figure 11:**
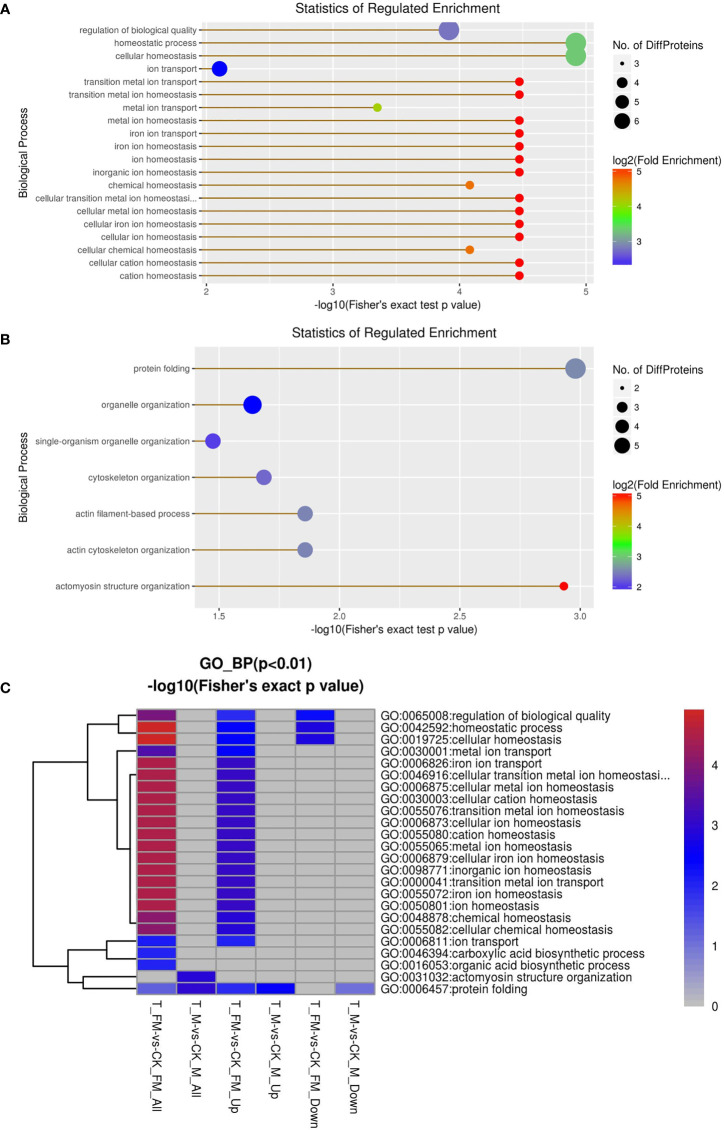
GO enrichment analysis for biological process in female **(A)** and male **(B)** adult *S. japonicum* specimens.

**Figure 12:**
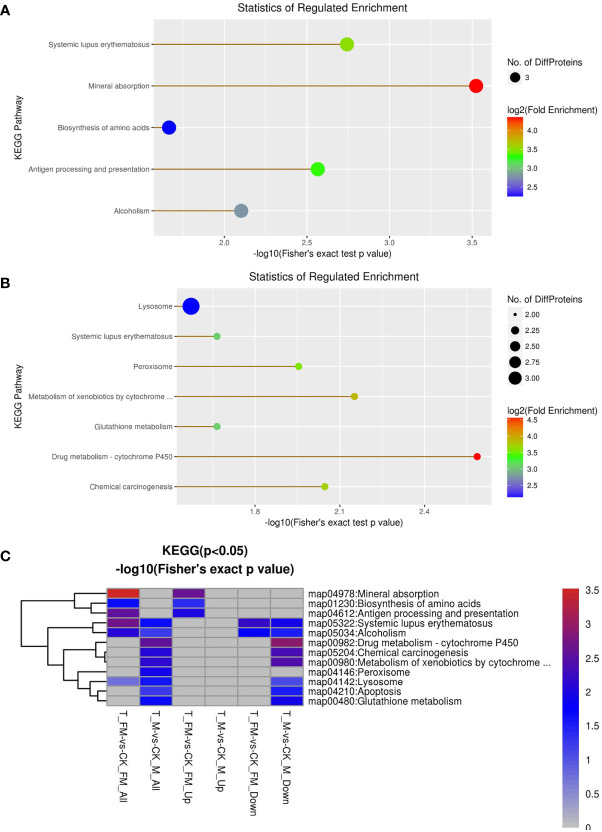
GO enrichment analysis for KEGG in female **(A)** and male **(B)** adult *S. japonicum* specimens.

GO enrichment analysis was used for DEPs associated with molecular function, including oxidoreductase activity, ferroxidase activity, ferric iron binding, iron ion binding, transition metal ion binding, cysteine-type peptidase activity, glutathione transferase activity, peroxiredoxin activity, lipid binding, transferase activity, oxidoreductase activity, peroxidase activity, calcium-dependent phospholipid binding, peptidasTae activity, antioxidant activity and phospholipid binding (**Figures 10A, B**). The results revealed 29 DEPs from females and 31 DEPs from males among 1216 proteins. Fourteen DEPs and 12 DEPs were upregulated in females and males, respectively, while 15 DEPs and 19 DEPs were downregulated in females and males, respectively (**Figure 10C**). Subsequently, GO enrichment analysis was used for DEPs associated with biological processes, including cellular homeostasis, homeostatic processes and transition metal ion homeostasis (**Figures 11A, B**). The result s revealed 19 DEPs in females and 19 DEPs in males among 881 proteins. Eleven DEPs and 7 DEPs were upregulated in females and males, respectively, while 8 DEPs and 12 DEPs were downregulated in females and males, respectively (**Figure 11C**). KEGG enrichment analysis revealed 19 DEPs in females and 17 DEPs in males among 1009 proteins. The DEPs from females were involved in mineral absorption, systemic lupus erythematosus, antigen processing and presentation, alcoholism and biosynthesis of amino acids (**Figure 12A**). It is interesting that KEGG enrichment analysis revealed the DEPs from not males but females were involved in mineral absorption. Mineral absorption plays an important role in female adult *S. japonicum* specimens, and ferritin is an important DEP that regulates the transport and storage of the iron pool (**Figure 12**). Ferritin is a universal intracellular protein that stores iron and releases it in a controlled fashion and plays an important role in inorganic ion transport and metabolism (Botebol et al., 2015). Studies have shown that ferritin deficiency in myeloid compartments dysregulates host energy metabolism and increases susceptibility to *Mycobacterium tuberculosis* infection (Reddy et al., 2018). A recent study demonstrated that mitochondrial ferritin plays an important role in preventing neuronal damage by regulating iron metabolism and attenuating oxidative stress (You et al., 2016). Increased expression of mitochondrial ferritin provides new insights into the antioxidant role of ferritin in the brains of Alzheimer’s disease (AD) patients (Yang et al., 2015). A *Listeria monocytogenes* ferritin-like protein plays an essential role in acid stress tolerance (Milecka et al., 2015). Our study showed that ferritin plays an important role in the iron metabolic pathway.

In contrast, KEGG enrichment analysis of the DEPs from males were involved in signaling pathways of drug metabolism, metabolism, chemical carcinogenesis, the peroxisome, glutathione metabolism, systemic lupus erythematosus and the lysosome (**Figure 12B**). Ten DEPs and 5 DEPs were upregulated in females and males, respectively, while 9 DEPs and 12 DEPs were downregulated in females and males, respectively (**Figure 12C**). It is also interesting that KEGG enrichment analysis the peroxisome involved in males but females were involved in peroxisome.

The peroxisome is also one of the main enriched metabolic pathways. Peroxisomes are a type of organelle (microbody) found in all eukaryotic cells and are involved in the catabolism of fatty acids (Gabaldón, 2018), amino acids, polyamines and hydrogen peroxide (Bonekamp et al., 2009). Notably, peroxisomes are capable of participating in the biosynthesis of plasmalogens, which are critical for the normal function of the mammalian brain and lungs (Bonekamp et al., 2009). Our results indicated that peroxiredoxin 1 (PRDX1) (Cai et al., 2015; Gong et al., 2015; Mullen et al., 2015) and superoxide dismutase (SOD) play important roles in hydrogen peroxide metabolism by the antioxidant system. Further studies are needed to investigate the functions of these two proteins.

A total of 123 *S. japonicum* DEPs were identified; 68 DEPs were from female adult worms, and 55 DEPs were from male adult worms. The bioinformatics analysis revealed that the DEPs were mainly involved in transport and metabolism, signal transduction mechanisms, energy production and conversion, and chromatin structure and dynamics. Putative DEPs between female and male adult worms was summed up in **Tables 1**, **2**. Receptor expression-enhancing protein were found in both putative DEPs between female and male *S.japonicum* proteins (**Tables 1**, **2**), which involve in intracellular trafficking, secretion, and vesicular transport. The specific protein followed such as: Firstly, Acyl carrier protein is multiple function protein involve in down-regulation of energy production and conversion, lipid transport and metabolism, secondary metabolites biosynthesis, transport and catabolism. Secondly, Saposin-like, IPR008139 Saposin B, domain-containing protein is a novel protein in female *S.japonicum*, which previous report in human, rat and mouse (Dhakshinamoorthy et al., 2018). The lastly, Calcium-binding EF-hand, domain-containing protein is widely in several host involved in signal transduction mechanisms. Compare with important female parasite proteins, putative DEPs of male *S.japonicum* were identified in **Table 2**. Previous report Cell death-regulatory protein GRIM19 in human, mouses and bovine, here a novel Cell death-regulatory protein GRIM19 was found in male *S.japonicum* involve in energy production and conversion, cell cycle control, cell division and chromosome partitioning. Meanwhile, we also found a metallophosphoesterase protein which down-regulation of nucleotide transport and metabolism and lipid transport and metabolism. Furthermore, T-complex protein 1 subunit delta can either upregulation or downregulation in posttranslational modification, protein turnover, chaperones.

**Table 1:** Putative annotations of differentially expressed proteins (DEPs) of female *S.japonicum*.

Protein (FM)	Description OS=Schistosoma japonicum	T_FM-vs-CK_FM (Ratio)	Regulated-Stage
Chromatin structure and dynamics			
Q5DDU9	Histone H2B	1.2806	Up
C1LVH1	Histone H3	0.7715	Down
Q5DET3	Histone H4	0.7581	Down
Energy production and conversion			
C1L5E9	Acyl carrier protein	0.5345	Down
Amino acid transport and metabolism			
Q86EI1	Glutamine synthetase	1.3368	Up
H6QX60	Tyrosinase 1	0.6929	Down
Carbohydrate transport and metabolism			
C1LT10	Phosphoglycerate kinase	1.2553	Up
C1L9G8	Saposin-like,IPR008139 Saposin B,domain-containing protein:	0.7773	Down
C1LD38	Glyceraldehyde-3-phosphate dehydrogenase	0.4602	Down
Lipid transport and metabolism			
C1L9G8	Saposin-like,IPR008139 Saposin B,domain-containing proteinc	0.7773	Down
C1L5E9	Acyl carrier protein	0.5345	Down
Inorganic ion transport and metabolism			
Q5DCX4	Ferritin	1.3269	Up
C1L8P3	Voltage-dependent anion-selective channel protein 2	1.2775	Up
C1LRQ1	Ferritin	0.7790	Down
Secondary metabolites biosynthesis, transport and catabolism			
C1LRD4	Carbonyl reductase 1	1.2825	Up
C1L5E9	Acyl carrier protein	0.5345	Down
Translation, ribosomal structure and biogenesis			
Q5DDJ0	Ribosomal protein S10	0.8002	Down
C1L7X1	Seryl tRNA Synthetase	0.6876	Down
Transcription			
C1LMM2	Transcription factor BTF3	0.7516	Down
Posttranslational modification, protein turnover, chaperones			
C1LMQ6	Peptidylprolyl isomerase	1.5611	Up
C1LNQ7	Protein disulfide-isomerase	1.3438	Up
C1L625	Chaperonin containing TCP1, subunit 7 (Eta)	1.3209	Up
C7TZI9	Heat shock protein 90kDa alpha	1.7338	Up
C1LDI9	Legumain	1.9436	Up
C1L9K4	Thioredoxin	0.5497	Down
C1LDR6	Legumain	0.6657	Down
O18533	Preprocathepsin C	0.8206	Down
Extracellular structures			
C1L7M7	Innexin	1.3635	Up
Intracellular trafficking, secretion, and vesicular transport			
Q5DBJ1	Receptor expression-enhancing protein	1.3963	Up
C1LIH6	Translocating chain-associated membrane protein:	0.6374	Down
C1L9D7	Receptor expression-enhancing protein	0.8141	Down
Signal transduction mechanisms			
C1LYI9	Calcium-binding EF-hand, domain-containing protein	1.2475	Up
C1LYM5	Calcium-binding EF-hand,domain-containing protein	0.5771	Down

**Table 2:** Putative annotations of differentially expressed proteins (DEPs) of male S.japonicum.

Protein (Male)	Description OS=Schistosoma japonicum	T_M-vs-CK_M (Ratio)	Regulated-Stage
Energy production and conversion			
Q86FC0	Cell death-regulatory protein GRIM19	0.7724	Down
Cell cycle control, cell division, chromosome partitioning			
Q86FC0	Cell death-regulatory protein GRIM19	0.7724	Down
Amino acid transport and metabolism			
Q5DBC6	SJCHGC09313 protein	0.7859	Down
Nucleotide transport and metabolism			
C1LJ94	Metallophosphoesterase	0.7551	Down
Lipid transport and metabolism			
C1LGC4	Lamin B receptor	0.6122	Down
C1LJ94	Metallophosphoesterase	0.7551	Down
Translation, ribosomal structure and biogenesis			
Q5C193	Uncharacterized protein	1.4747	Up
C7TZS6	Lysyl-tRNA synthetase (Fragment)	1.2503	Up
C1L909	Ribosomal protein L38	0.7297	Down
Posttranslational modification, protein turnover, chaperones			
C1LB64	Tryparedoxin peroxidase	1.2311	Up
C1LMQ4	Peptidylprolyl isomerase	1.2005	Up
C1L4U7	Carboxypeptidase	1.2472	Up
C1L5N9	T-complex protein 1 subunit delta	1.3671	Up
C1L732	Cathepsin L-like proteinase	0.8923	Up
C1L8H7	Hypotherical protein	1.1398	Up
C7TZI9	Heat shock protein 90kDa alpha (Fragment)	1.6532	Up
O18533	Preprocathepsin C	1.5672	Up
O96072	Calpain	2.1812	Up
C1LV44	Tryparedoxin peroxidase	0.5283	Down
P08515	Glutathione S-transferase class-mu 26 kDa isozyme	0.8105	Down
C1L625	Chaperonin containing TCP1, subunit 7 (Eta)	0.7538	Down
C1LCI7	Glutathione S-Transferase	0.7546	Down
C1LI91	GTP-binding-protein	0.7793	Down
Q5BZH7	SJCHGC08025 protein (Fragment)	0.8296	Down
C1L8R0	Cathepsin B	0.8323	Down
C1LI13	T-complex protein 1 subunit delta	0.7106	Down
C1L5R3	N-acetyl galactosaminidase, alpha	0.7511	Down
General function prediction only			
Q5C164	Tetraspanin (Fragment)	1.9619	Up
Q5BS84	SJCHGC05502 protein (Fragment)	3.6141	Up
Q5C167	Tetraspanin	0.7008	Down
Q86EB6	Clone ZZD581 mRNA sequence	0.8220	Down
Signal transduction mechanisms			
Q3MJS5	SJCHGC06847 protein (Fragment)	0.8278	Down
C1LGC4	Lamin B receptor	0.6122	Down
Intracellular trafficking, secretion, and vesicular transport			
C1L652	Annexin A3 (Annexin III)	1.2091	Up
Q5DBJ1	Receptor expression-enhancing protein	1.7629	Up
C1L9D7	Receptor expression-enhancing protein	0.7602	Down
C1LI91	GTP-binding-protein	0.7793	Down
C1L7Y3	Annexin A13 (Annexin XIII)	0.7151	Down
Defense mechanisms			
B3W658	Uncharacterized protein	0.4453	Down
Cytoskeleton			
B5B7R6	Calponin	1.3795	Up
C1LND6	Putative dynein light chain	1.2530	Up
Q5DAC9	Tubulin alpha chain	0.8202	Down
O96410	Calponin	0.4299	Down
C1LJL3	Actin-like protein 3	0.7085	Down

In the present study, we used iTRAQ to identify DEPs in adult schistosomes in mice after development in two *S. japonicum* intermediate hosts, *O. hupensis* and *O. weishan.* The bioinformatics analysis showed that the DEPs differed significantly between females and males after development in two intermediate hosts, and some DEPs might play important roles in signaling pathways and nutrient metabolism. Comparative proteomics provided new insights into the developmental mechanisms of schistosomes and the relationships between schistosomes and their hosts.

## Materials and methods

### *Oncomelania* snails

*O. weishan* was kept in the southern foothills of Dushan Island in Weishan Lake in Shandong Province. After 13 years of breeding on Dushan Island and 12 generations of snails, the shell shape and population of the snails changed. Adult *O. hupensis* specimens were collected from the Yangzhou section of the Changjiang River.

### Polymerase chain reaction and sequence

The total genomic DNA of *O.weishan* snails was extracted using a standard procedure. Polymerase chain reaction (PCR) was used to generate a fragment spanning ITS1-5.8S-ITS2 between the forward primer 5.8SF (5’- ATTGAACGGTTTAGTGAGGTCC -3’) and the reverse primer 5.8SR (5’-CATTCCCAAACAACCCGACTC -3’) based on available GenBank sequences. The PCR protocols were 94°C for 3 min followed by 30 cycles of 94°C for 30s, 58°C for 30s, and 72°C for 90 s and then a final elongation step at 72°C for 10 min. The amplified products were purified on a 1.0% agarose gel using the DNA gel extraction kit. The purified PCR product was then sequence and deposited in the GenBank database under accession numbers.

### Sample preparation

All Oncomelania snails were removed from the feeding plate of the 25°C incubator. A 50 ml beaker was added to the room temperature laboratory environment. After adding 40 ml of dechlorinated water for 4 hours, a large number of cercariae were found by anatomic microscopy and counted in 10 consecutive fields with a fungus ring on 6 × 10 cm cover slips. The release of the two groups of cercariae from the *Oncomelania* snails was then observed. In addition, the cercariae and the water covering them were overturned on the bare abdomens of the mice to infect each mouse with 20 cercariae, and the mice were allowed to rest for 10 min. Fifty days after infection, two groups of mice (T: *O. weishan* and CK: *O. hupensis*) were randomly numbered, and the mice were killed by excision of the eyeballs and bleeding of the cervical spine. Then, the abdominal cavity, intestines, mesentery, liver, spleen, kidney and other organs were removed from the abdominal cavity. Female and male adult schistosomes were separated from the mesenteric vein by ophthalmic tweezers and counted in physiological saline. We analyzed the protein expression profiles of the two groups of adult schistosomes. Ten snails per group were collected and pooled to eliminate the effects of individual differences. Total proteins were extracted from each developmental sample using a phenol extraction procedure, and the protein concentrations were determined using the Bradford colorimetric method. The animal use protocol and mouse procedures complied with the guidelines of the Association for Assessment and Accreditation of Laboratory Animal Care International.

### Proteolysis

According to the protein quantitation and SDS-PAGE results, 100 µg samples were adjusted to equal volumes with 8 M urea buffer. The final concentration of 10 mM DTT was added, the samples were incubated for 45 min at 37°C; the final concentration of 25 mM IAM was then added, and the samples were incubated at room temperature for 55 min. Then, 100 mM TEAB diluted to achieve a urea concentration below 2 M after enzymatic hydrolysis was added, and trypsin (1:50 dilution) was added overnight at 37°C; trypsin was then added to achieve a mass ratio of 1:100 prior to the second enzymolysis at 37°C for 4 hours.

### iTRAQ labeling and peptide fraction

iTRAQ labeling was performed using iTRAQ Reagent 4-plex according to the manufacturer’s protocol. Proteins from the worms in the four groups were labeled with reagents 113, 115, 117 and 119. Four independent repetitions were performed for each group. The labeled peptide was separated into 10-20 components by HPLC on a C18 column. Forty peptide fractions were collected, dried in a vacuum and separated into 20 components by mass spectrometry.

### Mass spectrometric analysis (LC-MS/MS)

Twenty components were dissolved in 20 µl of nano LC A solution. A 2 µl sample was injected into the column gradient and eluted, analyzed and identified by Orbitrap Elite mass spectrometry. The sample was analyzed by a Thermo Nano LC 1000 high-performance liquid chromatography (HPLC) system, and an Acclaim PepMap^®^ 100 C18, 3 µm, 100 Å (75 µm × 2 cm) sample column and an Acclaim PepMap^®^ RSLC C18, 2 µm, 100 Å (50 µm × 15 cm) analysis column were applied in the mass spectrometric analysis.

### Database searching and bioinformatics

Proteome Discoverer 1.3 (Thermo Scientific) software was used to transform the original map file (Orbitrap Elite) into an mgf file, and the data were submitted to the MASCOT 2.3.0 server. The server performs database retrieval, and a library file (.dat article) was used to achieve an FDR<0.01 standard to obtain highly credible qualitative results. According to the Gene Ontology (GO) annotation information for the identified proteins, we calculated the number of DEPs for each GO term at level 2.

## Data availability statement

The mass spectrometry proteomics data have been deposited to the ProteomeXchange Consortium (http://proteomecentral.proteomexchange.org) via the iProX partner repository with the accession number: PXD035090.

## Ethics statement

All protocols related to animals were carried out based on the guidelines of the Association for Assessment and Accreditation of Laboratory Animal Care International.

## Author contributions

FM, YW and JK performed the experiments, participated in the design of the study. GL and FM designed, drafted and participated the revision of the manuscript. KY, WL, CX, FZ, XW participated in the animal experiment. HY and CW participated in collection of oncomelania snails. GY and CZ provide suggestions for designed the experiments. All authors read and approved the final manuscript. All persons who have made substantial contributions to the work reported in the manuscript. All authors contributed to the article and approved the submitted version.

## Funding

This study was supported by the Science and Technology Development Plan of Shandong Medical and Health (No.2003-09) and Linyi University High-level Talent Funding Support (No.Z6122016). We would like to thank Monitor Helix MH BioTech Co.,Ltd provide for proteomic analysis.

## Conflict of interest

The authors declare that the research was conducted in the absence of any commercial or financial relationships that could be construed as a potential conflict of interest.

## Publisher’s note

All claims expressed in this article are solely those of the authors and do not necessarily represent those of their affiliated organizations, or those of the publisher, the editors and the reviewers. Any product that may be evaluated in this article, or claim that may be made by its manufacturer, is not guaranteed or endorsed by the publisher.
