# Corrigendum: Comparative proteomics analysis of *Schistosoma japonicum* developed in different *Oncomelania* snails as intermediate hosts

**DOI:** 10.3389/fcimb.2022.1100710

**Published:** 2022-12-16

**Authors:** Gongzhen Liu, Feng Miao, Yongbin Wang, Jingxuan Kou, Kun Yang, Wei Li, Chunrong Xiong, Fengjian Zhang, Xinyao Wang, Haoyun Yan, Changyin Wei, Changlei Zhao, Ge Yan

**Affiliations:** ^1^ College of Agriculture and Forestry, Linyi University, Linyi, Shandong Province, China; ^2^ Shandong Institute of Parasitic Diseases, Shandong First Medical University & Shandong Academy of Medical Sciences, Jining, Shandong Province, China; ^3^ Jiangsu Institutes of Parasitic Diseases, Wuxi, Jiangsu Province, China; ^4^ Fourth Hospital of Weishan, Jining, Shandong Province, China; ^5^ Shandong Weishan Center for Disease Prevention and Control, Jining, Shandong Province, China

**Keywords:** *Schistosoma japonicum* (*S.japonicum*), schistosomiasis, isobaric tags for relative and absolute quantification (iTRAQ), differentially expressed proteins (DEPs), *oncomelania hupensis* (*O.hupensis*), *oncomelania weishan* (*O.weishan*)

**Incorrect Affiliation**

In the published article, there was an error in affiliation(s). Instead of “^1^Shandong Institute of Parasitic Diseases, Shandong First Medical University & Shandong Academy of Medical Sciences, Jining, Shandong Province, China; ^2^College of Agriculture and Forestry, Linyi University, Linyi, Shandong Province, China,”, it should be “^1^College of Agriculture and Forestry, Linyi University, Linyi, Shandong Province, China;^2^Shandong Institute of Parasitic Diseases, Shandong First Medical University & Shandong Academy of Medical Sciences, Jining, Shandong Province, China;”.

The authors apologize for this error and state that this does not change the scientific conclusions of the article in any way. The original article has been updated.

## Error in Author List

In the published article, there was an error in the author list, and author [Xin Liu] was erroneously included. The corrected author list appears below.

Correct author list: Gongzhen Liu^1,2^, Feng Miao^2*^,Yongbin Wang^2^, Jingxuan Kou^2^, Kun Yang^3^, Wei Li^3^, Chunrong Xiong^3^, Fengjian Zhang^3^, Xinyao Wang^3^, Haoyun Yan^4^, Changyin Wei^5^, Changlei Zhao^2^ and Ge Yan^2^

The authors apologize for this error and state that this does not change the scientific conclusions of the article in any way. The original article has been updated.

## Text Correction

In the published article, there was an error in Author Contribution. There was a spelling mistake in Author Contribution section, “designe” need to be revised as “designed”.

A correction has been made to Author Contributions. This sentence previously stated:

“GL and FM designe, drafted and participated the revision of the manuscript.”

The corrected sentence appears below: “GL and FM designed, drafted and participated the revision of the manuscript.”

